# Supplementary material for: Genome-Wide Identification and Expression Pattern Analysis of TIFY Family Genes Reveal Their Potential Roles in Phalaenopsis aphrodite Flower Opening
Source: Int J Mol Sci. 2024 May 16;25(10):5422. doi: 10.3390/ijms25105422 (PMC11121579; doi:10.3390/ijms25105422)
Supplement: Supplementary file 1 [file ijms-25-05422-s001.zip › Supplemental Figure.pdf]

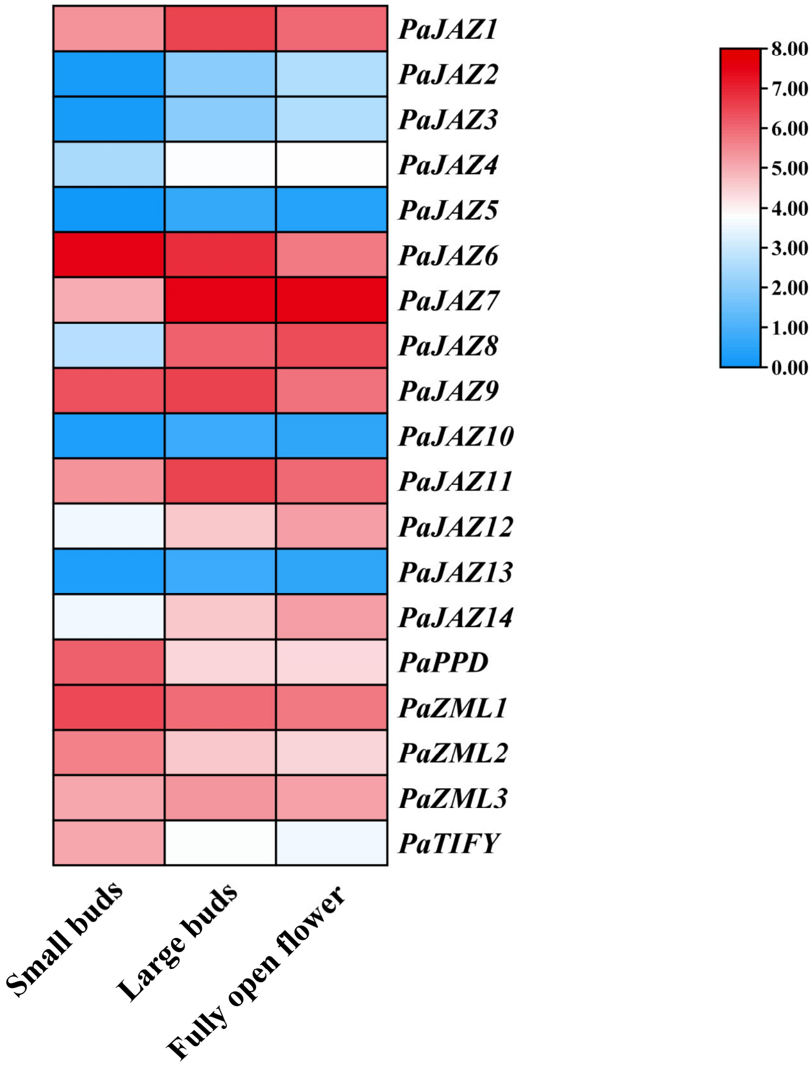

Figure S1 Heatmap of 19 *TIFY* genes in small buds, large buds, and fully open flower of *Phalaenopsis*. Red rectangles indicate high expression, while the blue rectangles represent low expression.

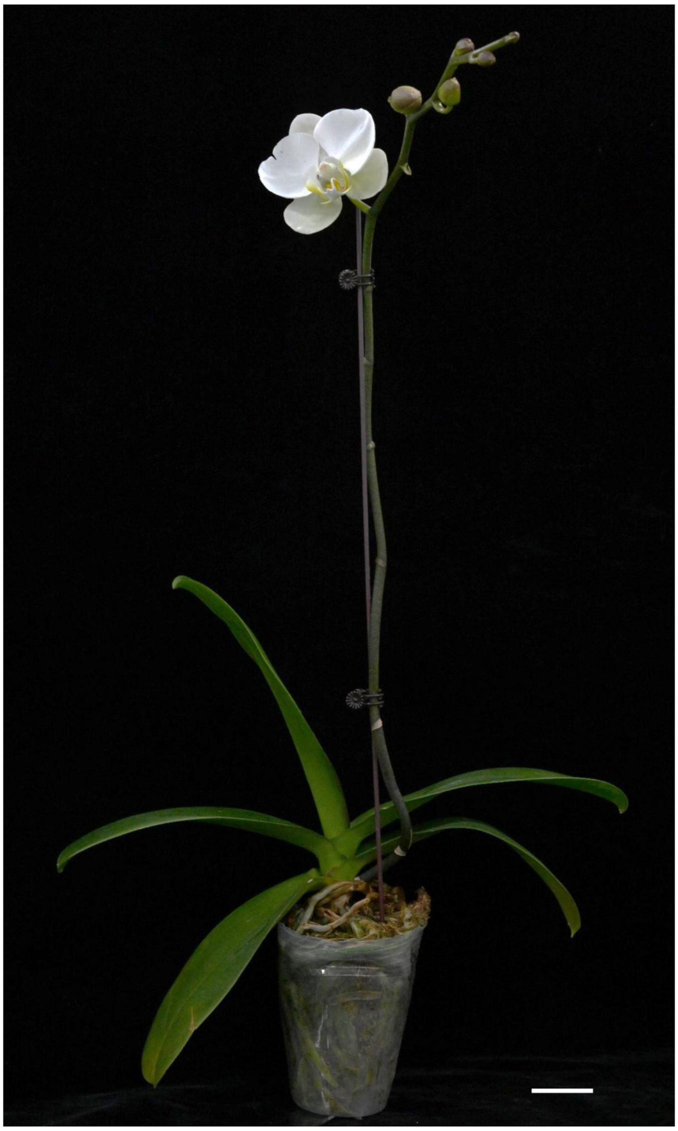

Figure S2 The whole flowering plant of *Phalaenopsis*. Scale bar = 3 cm.
